# Annotations

**Published:** 1895-07-13

**Authors:** 


### Annotations.

### Are Scientific Giants Extinct?

Huxley, we are assured on the highest journalistic authority, has left no successor. The King of British science is dead—shall we ever have another? The leading journal has grave doubts on this point. "Deep as was the dead man's interest in the positive discoveries of physical science . . . his dominant interest was in man," we are told; and, therefore, he was a king among men. "Is it the case," asks our contemporary, "that we have reached a stage in scientific progress when it will no longer be possible for a first-rate investigator to feel this wide human interest, and to devote the half of his time and of his mind to telling his fellow-men what is the ultimate meaning of the discoveries which he and others are making?" The mere asking of this question is tantamount to expressing the opinion that there is not now left a single man of the highest genius in the whole ranks of British science. We have, in fact, nothing but specialists left. Specialism may be, and is, inevitable. But the mere specialist is sometimes a very dangerous person. The specialist who is also a man of wide illuminating genius is always and everywhere frowned upon by the vast majority of his brother specialists. By what right does he stand upon a pedestal and give light to the world, whilst they, who also have light, can only shine in the narrower prison-house of their own specialism? There is one thing to be said of this attitude of mind: it destroys all the specialist's chances of recognition and honour among his fellow-men, and dooms him to perpetual obscurity. The great world never does homage to what it does not, at least to some extent, understand. But the great world cannot understand the specialism of the specialist, except by the specialist's goodwill and help. Let the specialist take Huxley for his ideal man of science. He may then save both himself and his specialism from the deadly dulness of the mere workshop, and help to perpetuate that race of scientific giants, of whom Huxley was the greatest.

### A New Lung for St. George the Martyr.

No space need be wasted in discussing the general proposition that every part of London ought to take every possible opportunity of creating for itself as many new lungs as it can. All that goes without saying. There is now an opportunity of opening out an excellent new lung, of great aërating capacity, in the most crowded and insanitary part of one of the most crowded and insanitary parishes in London, that of St. George the Martyr, Southwark. Falcon Court is an area which extends over about three acres. If were not covered to some extent with houses, it would make a very large paddock or small field. It contains a population of 757 human beings, to say nothing of cats, dogs, costers' donkeys, and other forms of organic life which are better imagined than named. But, as a matter of fact, the whole 757 inhabitants are crowded into about a third part of the area; that is, into one and a-quarter acre. There are, of course, the usual consequences of overcrowding to be seen in Falcon Court: the disease-rate is high, the death-rate is very high, and the destruction of helpless infant life is appalling. In short, Falcon Court is one of those blots on civilisation of which every intelligent County Councillor ought to be, and is, most sincerely ashamed. The foregoing is the briefest possible summarisation of the larger facts. What is to be done with Falcon Court? It can be purchased entire, with the houses on it, for £35,000—a very small sum for so large a space in the very centre of crowded London. The Medical Officer of Health for the parish, Dr. F. J. Waldo, is, and has been for three years, all in favour of purchasing the court outright, and turning it into a large open space, of which a great part shall be utilised as a playground for children. In our judgment, Dr. Waldo's view of the case is so much better than any other which can be maintained, that it ought to be adopted at once and by universal consent. It is whispered, though we cannot believe it to be true, that some of the wiseacres of the L.C.C., instead of agreeing to the pulling down of this vile rookery, and the conversion of the court into a three-acre playground, actually suggest the covering of some of the unoccupied space with warehouses. If Mr. Bumble were still alive, we might believe that such a suggestion had been made; but he is dead, and has left no successors, we trust. Undoubtedly, the County Council should waste no time in discussing this question, but secure this new lung for South London at all costs.

### Charity and Brains.

Charity with brains has been one of the greatest ameliorators of our latter-day over-driven centuries. Charity without brains threatens to bring all charity into disrepute, and to make it *anathema* to all reasoning men. The Rev. Canon Barnett has uttered a much-needed protest in the *Times* against one of the many forms of charity without brains. "A day in the country or at the seaside" for the children of our crowded London streets sounds altogether delightful to the grown-up workers and inhabiters of the same or other streets. And so it would be if the little ones could be placed at the seaside without the preliminary journey, and in just such numbers and under such care as to secure for them abundance of play, a few little careful dippings of hot and tired feet in the sea, regular supplies of digestible food and drink, and a sweet little bed at the end of a perfect day; and all without the dreadful journey home again by train. This is what the inexperienced town man who pays for these days in the country imagines them to be. Never was delusion more complete. Undoubtedly, the day in the country or at the seaside, accompanied as it is by a journey to the train, a long, dreary, crowded, and draughty ride in the train, a spiritless and miserable apology for play at the seaside, irregular and injurious eating and drinking, exposure of the little ones to the hot sun, the long and miserable return journey by train after every particle of nerve energy has been exhausted by fatigue, heat, and thirst: all this constitutes in its totality one of the most miserable attempts at helping town children to happiness and health that the most brainless philanthropy could possibly invent. Canon Barnett suggests the remedy; an admirable one. Send the children by small batches into the country, not for a day, but for a week, or even two weeks; and let them live in the labourers' cottages. They will then enter upon a really new life. The woods, the fields, the birds, the flowers, everything will be delightful. They will sleep in good air, their blood will be purified, their appetites quickened, their spirits raised; they will make a new physiological start in life. Canon Barnett's method is "charity with brains." Let every man and woman who has brains support him, and stamp out the senseless philanthropy which he so justly condemns.

